# Molecular Characterization of the Onset and Progression of Colitis in Inoculated Interleukin-10 Gene-Deficient Mice: A Role for PPAR*α*

**DOI:** 10.1155/2010/621069

**Published:** 2010-06-30

**Authors:** Bianca Knoch, Matthew P. G. Barnett, Janine Cooney, Warren C. McNabb, Diane Barraclough, William Laing, Shuotun Zhu, Zaneta A. Park, Paul MacLean, Scott O. Knowles, Nicole C. Roy

**Affiliations:** ^1^Food, Metabolism & Microbiology Section, Food & Textiles Group, AgResearch Grasslands, Tennent Drive, Palmerston North 4442, New Zealand; ^2^Institute of Food, Nutrition & Human Health, Massey University, Tennent Drive, Palmerston North 4442, New Zealand; ^3^Plant & Food Research, East Street, Hamilton 3214, and 120 Mt Albert Road, Sandringham, Auckland 1025, New Zealand; ^4^Food & Textiles Group, AgResearch Grasslands, Tennent Drive, Palmerston North 4442, New Zealand; ^5^Riddet Institute, Massey University, Palmerston North 4442, New Zealand; ^6^Faculty of Medical and Health Sciences, The University of Auckland, 85 Park Road, Grafton, Auckland 1023, New Zealand; ^7^Bioinformatics, Mathematics & Statistics Section, AgResearch Grasslands, Tennent Drive, Palmerston North 4442, and AgResearch Ruakura, East Street, Hamilton 3240, New Zealand

## Abstract

The interleukin-10 gene-deficient (*Il10* ^−/−^) mouse is a model of human inflammatory bowel disease and *Ppara* has been identified as one of the key genes involved in regulation of colitis in the bacterially inoculated *Il10* ^−/−^ model. The aims were to (1) characterize colitis onset and progression using a histopathological, transcriptomic, and proteomic approach and (2) investigate links between PPAR*α* and IL10 using gene network analysis. Bacterial inoculation resulted in severe colitis in *Il10* ^−/−^ mice from 10 to 12 weeks of age. Innate and adaptive immune responses showed differences in gene expression relating to colitis severity. Actin cytoskeleton dynamics, innate immunity, and apoptosis-linked gene and protein expression data suggested a delayed remodeling process in 12-week-old *Il10* ^−/−^ mice. Gene expression changes in 12-week-old *Il10* ^−/−^ mice were related to PPAR*α* signaling likely to control colitis, but how PPAR*α* activation might regulate intestinal IL10 production remains to be determined.

## 1. Introduction

The interleukin-10 gene-deficient (*Il10* ^−/−^) mouse is a well-established model of human inflammatory bowel disease (IBD) and used to study the complex host-environment (e.g., diet, bacteria) interactions and the action of potential therapeutics [1]. *Il10* ^−/−^ mice develop a Crohn's disease (CD)-like colitis when exposed to commensal bacteria, whereas no colitis is observed in the wildtype and germ-free *Il10* ^−/−^ mice, which suggests an important role for IL10 in controling tolerance towards commensal bacteria and in preventing colitis in these mice [2, 3]. IL10 signals through Janus kinase 1/signal transducer and activator of transcription 3 and p38 mitogen-activated protein kinase-dependent pathways to induce suppressor of cytokine signaling-mediated [4] or heme oxygenase 1-dependent [5] anti-inflammatory mechanisms. The immunopathology in *Il10* ^−/−^ mice represents a T-helper cell type 1 (Th1)- and Th17-polarized inflammation with high colonic expression of interferon gamma (IFN*γ*) as the main Th1-derived pro-inflammatory cytokine and IL17 [6, 7]. 

Both the severity and time-course of colitis in *Il10* ^−/−^ mice are influenced by the inbred strain background when maintained under the same conditions. The colitis in *Il10* ^−/−^ mice on the C57BL/6J background is mild to moderate [6], compared with when the *Il10* ^tm1Cgn^ allele is bred into the 129/SvEv, C3H/HeJBir, or C3H.SW background strains, where colitis is severe and progressive [8–10]. This suggests that other genes or gene interactions particular to the genetic background of each strain modify the development of colitis. Intestinal inflammation also develops more consistently when *Il10* ^−/−^ mice (C57BL/6J background) raised under conventional conditions are inoculated with a mixture of pure *Enterococcus* isolates (*E. faecalis* and *E. faecium*) alone, or combined with conventional intestinal flora derived from healthy C57BL/6J mice [11]. The timeline of colitis development is unknown in these bacterially inoculated *Il10* ^−/−^ (C57BL/6J) mice.

Studies to date have mostly applied transcriptomic [11–14] and proteomic [15] methods separately to intestinal tissue samples of various murine models of experimental colitis and of IBD patients. It is important to integrate gene and protein expression data to get a more comprehensive understanding of phenotypic changes. A previous study showed that conventional non-inoculated *Il10* ^−/−^ mice (C57BL/6J background) developed increasing colonic inflammation, peaking at 12 weeks of age [16]. 

The first hypothesis of this study was that distinct gene and protein expression patterns could be defined in non-inflamed (7 weeks of age) and colitic (12 weeks of age) *Il10* ^−/−^ mice. Since the time-course of colitis in the bacterially inoculated *Il10* ^−/−^ mouse model (C57BL/6J background) used by Roy et al. [11] is undefined, the first aim was to characterize colitis onset and progression in this model and to extend previous studies [11–15] by using a combined histopathological, transcriptomic, and proteomic approach. 

Gene network analysis identified the *Ppara* gene as one of the key genes with decreased expression levels associated with severe colitis when comparing bacterially inoculated *Il10* ^−/−^ and C57 mice [17]. A critical role for PPAR*α* in regulating inflammation was first identified by Devchand et al. [18] who observed a prolonged inflammatory response in Ppara^−/−^ mice. PPAR*α*-ligands showed anti-inflammatory effects in experimental colitis including the *Il10* ^−/−^ mouse model [19, 20]. Based on these studies [17–20], the second hypothesis was that PPAR*α* and IL10 signaling pathways are interlinked during colitis. The secondary aim was to investigate the involvement of PPAR*α* regulation in *Il10* ^−/−^ mice before and after colitis onset using gene network analysis.

## 2. Methods and Materials

### 2.1. Animals and Induction of Colitis

A total of 30 male *Il10* ^−/−^ (C57BL/6J background, formal designation B6.129P2-*Il10*<tm1Cgn>/J) and 30 male C57 control (C57BL/6J) mice were obtained from The Jackson Laboratory (Bar Harbor, Maine, USA). For convenience and consistency in reporting, their age was defined as 35 days or 5 weeks of age at the start of the study. Mice were individually housed in standard shoebox size cages containing untreated wood shavings (Hi Tech Security Disposals Ltd., Auckland, New Zealand) and maintained under conventional conditions with a temperature of approximately 22°C, 50% relative humidity, and a 12-hour light-dark cycle. 

After 4 days, all mice were inoculated orally with a mixture of pure *E. faecalis* and *E. faecium* strains and complex intestinal flora derived from healthy C57BL/6J mice raised under conventional conditions to obtain a more consistent and reproducible intestinal inflammation, as described previously [11].

### 2.2. Experimental Design

The objective of this experiment was to study the onset and progression of colitis and associated changes in gene and protein expression in bacterially inoculated *Il10* ^−/−^ mice as a model for future nutrigenomics studies to explore the effects of nutrition on IBD pathophysiology. Mice were randomly assigned to 5 sampling groups (7, 8.5, 10, 12, and 14 weeks of age). The mice had free access to water and were fed an AIN-76A standard powder diet prepared in-house. The diet composition has been described previously [11]. Throughout the experimental period, dietary intake was estimated daily by weighing uneaten food and adjusted to equal the mean amount of food consumed by the *Il10* ^−/−^ mice on the previous day. All mice were weighed thrice a week and carefully monitored for disease symptoms (weight loss, soft feces, inactivity).

Tissue sampling was performed at 7, 8.5, 10, 12, and 14 weeks of age. Mice were euthanized by CO_2_ asphyxiation and cervical dislocation, and cardiac puncture was then performed. There was a fast-feed period prior to sampling as described previously [17], and the intestine was isolated and cut open lengthwise. One piece of each intact intestinal section (duodenum, jejunum, ileum, and colon) was stored at room temperature in 10% phosphate-buffered formaldehyde for histopathological assessment; another was immediately frozen in liquid nitrogen and kept at −80°C for gene and protein profiling.

### 2.3. Histology

The histopathological assessment of the full thickness intestinal sections (duodenum, jejunum, ileum or colon) and the scoring method have been described previously [17]. This method produces a histological injury score (HIS) for each sample. It is the sum (total HIS) of principal histological aspects (inflammatory cell infiltration, tissue destruction, and tissue repair). A total HIS score of each intestinal section from 0–3 was regarded as no inflammation, from 4–6 as moderate inflammation and ≥7 as severe inflammation.

### 2.4. RNA Isolation, Microarray Hybridization, and Analysis

RNA isolation and microarray hybridization have been described previously [17]. The microarray experiment used 15 arrays based on the histopathological assessment of the colon: three colon samples of *Il10* ^−/−^ mice at 7 weeks of age (no colitis), four of *Il10* ^−/−^ mice at 12 weeks of age (moderate colitis), four of C57 mice at 7 weeks of age (no colitis), and four of C57 mice at 12 weeks of age (no colitis). At 7 weeks of age, one *Il10* ^−/−^ mouse died of unknown causes and two others were already showing moderate signs of colon inflammation and were thus excluded. Each individual RNA sample was hybridized with a reference sample onto the array. Array data were submitted to the Gene Expression Omnibus, accession number GSE17990.

Statistical analysis and quality assessment of the microarray data were performed using linear models for microarray analysis (limma) within the Bioconductor framework as described previously [17]. All arrays passed the quality control and were included in the analyses. Intensity ratios for all microarray spots were normalized using a local linear regression analysis (LOESS) to remove the effect of systematic variation in the microarrays and no background correction was necessary due to homogeneous hybridization. The normalized array data of each time point were log_2_-transformed and averaged. For each comparison of interest (*Il10* ^−/−^ versus C57 mice 7 weeks and *Il10* ^−/−^ versus C57 mice 12 weeks), a list of differentially expressed genes was generated.

### 2.5. Quantitative RT-PCR

Quantitative RT-PCR (qRT-PCR) has been described previously [17]. Ten genes (ATP-binding cassette subfamily B member 1, *Abcb1A*; aldehyde dehydrogenase 1 family member A1, *Aldh1A1*; carboxylesterase 2, *Ces2*; fatty acid binding protein 2, *Fabp2*; insulin-like growth factor binding protein 5, *Igfbp5*; interleukin 1 beta, *Il1B*; matrix metallopeptidase 13, *Mmp13*; *Ppara,* sterol regulatory element binding protein 1, *Srebf1* and sulfotransferase family 1A phenol-preferring member 1, *Sult1A1*) were used for quantification and microarray verification. Genes were selected to include both significantly and non-significantly regulated genes pertaining to pathways affected by inflammation, for example, those related to detoxification, transport, immunity, or metabolism. The primer sequences for target and reference genes are available upon request. The mRNA expression of all genes reported was normalized to calnexin (*Canx*) gene expression.

### 2.6. Protein Isolation, LC-MS Analysis of Peptides, MS/MS Data Processing, and Analysis

The protein-containing lower layer of the same TRIzol processed sample from which RNA was derived was used for protein isolation and further identification by liquid chromatography and tandem mass spectrometry (LC-MS/MS). The protein pellets stored in 0.3 M guadinine hydrochloride in 95% ethanol were processed according to manufacturer's instructions (TRIzol protocol, Invitrogen) through two washes and a final ethanol wash, and then allowed to air-dry. Resolubilization buffer (7 M urea (BioRad), 2 M thiourea (Sigma), 4% CHAPS (BioRad), 40 mM Tris (Invitrogen)) was added and samples incubated at 22°C, 600 rpm, overnight in a Thermomixer (Eppendorf). After centrifugation, supernatants were used to determine protein concentration using the Bradford protein assay with bovine serum albumin as a standard [21]. Volumes of samples that required pooling were calculated to produce total aliquots of 50 *μ*g per treatment. Gels were run as duplicate biological replicates using the same samples as described for the microarray design. The 50 *μ*g total protein of treatment and control samples were labeled with 200 pmol of cyanine-2 and cyanine-5 dyes (GE Healthcare, Uppsala, Sweden), respectively, as described by the manufacturer. The labeled treatment and control sample were combined to make 100 *μ*g protein and run on Immobiline Drystrips (GE Healthcare, 18 cm, pH 3–11 nonlinear) in an equal volume of 7 M urea, 2 M thiourea, 4% CHAPS, a few grains of Bromophenol Blue (Sigma), 2% pH 3–11 NL IPG buffer (GE Healthcare), and 65 mM DTT buffer (Sigma) to separate the proteins in the first dimension. The first dimension, equilibration, and second dimension were performed as previously described [22]. Precision Plus protein standard plugs (BioRad) were used as molecular weight markers. Immediately after electrophoresis, the gels were washed in double-distilled H_2_O and visualized using a Typhoon (TM) 9400 imager (GE Healthcare). The cyanine-2 images were scanned using a 488 nm laser and a 520 nm band pass 40 emission filter, whereas the cyanine-5 images were scanned using a 633 nm laser and a 670 nm band pass 30 emission filter. All gels were scanned at a resolution of 200 *μ*m, and analyzed using Phoretix 2D Evolution software (Nonlinear Dynamics). Staining was performed by placing gels into modified Neuhoff colloidal Coomassie stain (17% ammonium sulphate, 3% phosphoric acid, 34% methanol, 0.1% Coomassie G-250) [23], after which gels were dried on glass plates at room temperature under cellophane and stored.

Differentially expressed proteins were only flagged as significant where the fold abundance for each biological replicate changed in the same direction and either both gave a value either <−1.5- or >1.5- fold, or where one biological replicate gave a value <−2-or >2-fold and the other biological replicate gave a value <−1.3 or >1.3. Significant protein spots were excised from the dried gels, rehydrated in deionized water, and digested with trypsin. Briefly, 25 mM ammonium bicarbonate in 50% acetonitrile was added to the gel pieces which were then incubated in a Thermomixer (1400 rpm, 22°C, 10 minutes) with up to two repeats of this step, depending on the density of the original staining. The gel pieces were then dried in a vacuum centrifuge (Speedyvac) and rehydrated at room temperature in a trypsin/HCl mix (20 *μ*L trypsin of a stock made from 25 *μ*g vial of Roche modified trypsin (sequencing grade) in 50 *μ*L 1 mM HCl (BDH), 200 *μ*L NH_4_HCO_3_ (Sigma) pH 8.0, 10 *μ*L 100% acetonitrile (BDH)). Following overnight incubation in the Thermomixer (37°C, 600 rpm), 30 *μ*L of 5% formic acid (Pierce) in 50% acetonitrile (Sigma) was added to the gel pieces which were sonicated for 5 minutes. The samples were briefly centrifuged and the supernatent removed which was repeated twice. Two additional extractions in formic acid/acetonitrile were repeated and pooled. Recovered peptides were concentrated by reducing the final volume of the extracts to approximately 10 *μ*L in a vacuum centrifuge, followed by resuspension to a volume of 20 *μ*L with formic acid/acetonitrile. The peptide solutions were stored at −20°C until MS was performed.

Tryptic peptides were separated and analyzed using an Ettan multidimensional liquid chromatography system (GE Healthcare) coupled to an LTQ linear ion trap mass spectrometer with a nanospray ionisation interface (ThermoQuest, Finnigan, San Jose, CA, USA). Samples (2 *μ*L) were injected onto a 300 *μ*m ID × 5 mm trap column (Zorbax 300-SB C18) for in-line desalting and separated on a nanoscale reverse phase chromatography column 75 *μ*m ID × 150 mm, 3 *μ*m (LC Packings, San Francisco, CA, USA) in high-throughput configuration at 280 nL/minute with a linear gradient from 0 to 60% B over 50 minutes (A: 0.1% formic acid; B: 84% acetonitrile and 0.1% formic acid). Data were acquired using a top 3 experiment in data-dependent mode with dynamic exclusion enabled.

MS/MS data were analyzed using TurboSEQUEST protein identification software [24, 25] and spectra were searched against the NCBI (National Center for Biotechnology Information) *Mus musculus* database. Modifications were set to allow for the detection of oxidized methionine (+16) and carboxyamidomethylated cysteine (+57). The criteria used for a positive peptide identification for a doubly charged peptide were a correlation factor (XCorr) >2.0, a delta cross-correlation factor (dCn) >0.1 (indicating a significant difference between the best match reported and the next best match), and a high preliminary scoring (Sp). For triply charged peptides the correlation factor threshold was set at 2.5. All matched peptides were confirmed by visual examination of the spectra.

### 2.7. Bioinformatics Analysis of Pathways and Functions

IPA (Version 7.0, Ingenuity Systems Inc., Redwood City, CA, USA) was used for pathway, network, and functional analyses of differentially expressed probes in the microarray dataset as described previously [17] and of differentially expressed proteins. EASE (software version 2.0, National Institutes of Health, USA) was used to identify enriched biological themes within gene lists using GO category over-representation analysis [26]. A stringent set of gene probes differentially expressed according to the microarray analysis were uploaded into EASE along with a list of all genes on the microarray to test for over-representation of annotation classes. An EASE score (adjusted Fisher's exact test for statistical significance) was calculated for likelihood of over-representation of hierarchical categories based on biological processes, molecular functions, and cellular components using the GO public database. Gene categories with an EASE score <0.05 and an FDR or *q* < 0.05 were considered to be significantly over-represented. The data files containing gene and protein identifiers (gene and protein accession number) and the corresponding changes in expression levels were uploaded into the IPA program. Genes and proteins from the dataset that satisfied the cut-off criteria of FC ≥ 1.5 (up- or down-regulated), FDR or *q* < 0.05, and FC ≥ 1.5, respectively, were considered for analyses. Pathways were considered to be affected by the development of colon inflammation when the probability value calculated by the Fisher's exact test was <0.01 and where at least 20% of the genes from a particular pathway were differentially expressed in the microarray dataset.

### 2.8. Statistical Analysis

All statistical analyses (body weight, dietary intake, HIS, and qRT-PCR data) were performed using ANOVA in GenStat (10th edition, VSN International, Hemel Hempstead, UK), on log-transformed data where necessary in cases of unequal variances. A probability value of less than 0.05 was considered as significant while a probability value greater than 0.05 but lower than 0.10 was considered a trend.

## 3. Results

### 3.1. Animal Body Weight and Dietary Intake

There was no difference between *Il10* ^−/−^ and C57 mice in terms of average body weight at the beginning of the experiment (16.5 ± 0.3 versus 16.9 ± 0.2 g, Table 1). During the course of the experiment, *Il10* ^−/−^ mice gained weight more slowly (with a loss in body weight observed between 12, and 14 weeks of age) than C57 mice, resulting in a lower average body weight at 7, 8.5, 10, 12, and 14 weeks of age when compared to the C57 mice. This was significant (*P* < 0.05) at 10, 12, and 14 weeks of age. Dietary intake was not different between *Il10* ^−/−^ and C57 mice at any time point in the study (Table 1).

### 3.2. Development and Characterization of Intestinal Inflammation

**Figure 1:**
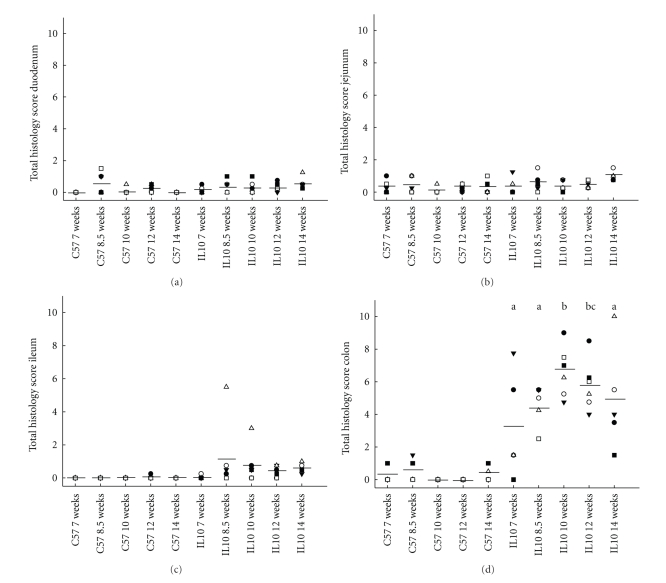
Total histological injury score for duodenum, jejunum, ileum, and colon sections of *Il10* ^−/−^ and C57 mice at 7, 8.5, 10, 12, and 14 weeks of age. The individual scores and the average score (—) of each sampling group are shown. Statistical analysis was performed only for total colon HIS of *Il10* ^−/−^ mice and different letters mean that total colon HIS differs compared to 7-week-old *Il10* ^−/−^ mice (a, b, *P* < 0.05 and c, 0.05 < *P* < 0.1).

Histological analysis showed that the average total HIS in *Il10* ^−/−^ mice was highest in the colon, and only two mice displayed moderate inflammation in the ileum. No signs of inflammation were observed in the duodenum or jejunum of *Il10* ^−/−^ mice or in any of the different intestinal sections of C57 mice (Figure 1). Therefore, statistical analysis was performed on the colon tissue of *Il10* ^−/−^ mice. There was a significant difference in the average total HIS in the colon between *Il10* ^−/−^ and C57 mice at 8.5, 10, 12 and 14 weeks of age. Two *Il10* ^−/−^ mice at 7 weeks of age showed initial signs of colon inflammation, but not the other three mice (Table 2). Most *Il10* ^−/−^ mice developed moderate colitis already by 8.5 weeks of age. It became apparent that the average total colon HIS of *Il10* ^−/−^ mice increased over time peaking between 10 and 12 weeks of age, and a decrease between 12 and 14 weeks of age.

**Figure 2:**
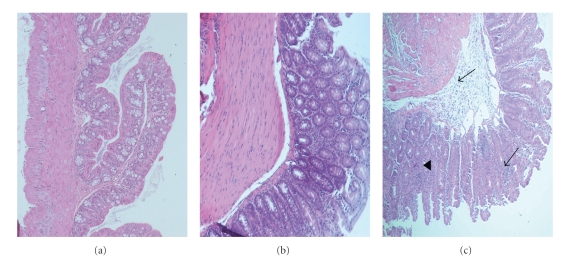
Hematoxylin-eosin-stained colon sections of *Il10* ^−/−^ and C57 mice. (a) Colon section (×20) from a non-inflamed C57 mouse at 12 weeks of age. (b) Colon section (×100) from an *Il10* ^−/−^ mouse at 7 weeks of age with no inflammation. (c) Moderate to severe inflamed colon section (×40) from an *Il10* ^−/−^ mouse at 12 weeks of age. Lesions involve most of the colon section with mainly monocyte and neutrophil infiltration (←), crypt abscesses, and loss of crypt and goblet cells (◂).

Colitis was mainly characterized by inflammatory cell infiltration (monocytes and neutrophils) but also featured tissue destruction (crypt loss and oedema) and tissue repair (hyperplasia). The colon of *Il10* ^−/−^ mice was highly inflamed by 10 weeks of age and showed moderate inflammation by 12 and 14 weeks of age (Figure 1). The total colon HIS in 10-week-old *Il10* ^−/−^ mice was significantly higher (*P* < 0.05) and a trend was observed at 12 weeks of age (*P* = 0.07) compared to 7-week-old *Il10* ^−/−^ mice. There was also less variability in the individual colon HIS at 8.5, 10, and 12 weeks compared to 7 and 14 weeks of age. The inflammatory lesions were transmural involving most layers of the intestinal wall. There was thickening of the mucosal layer and formation of crypt abscesses with loss of goblet cells (Figure 2).

### 3.3. Inflammation-Induced Changes in Expression Profiles

**Figure 3:**
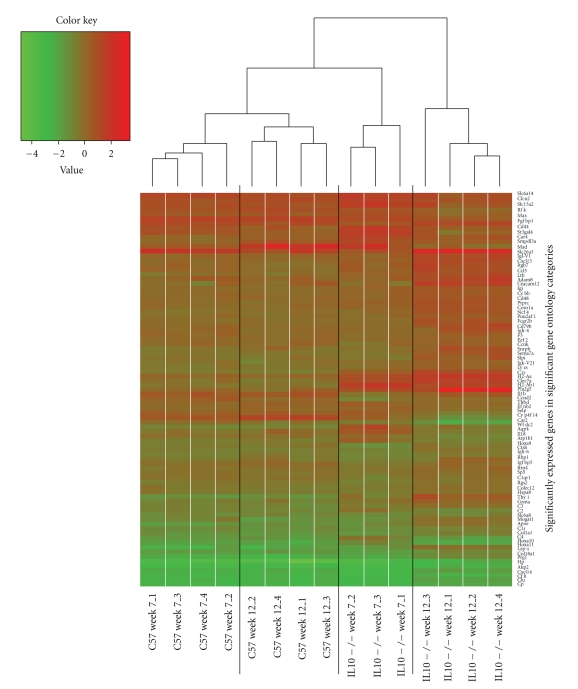
Genes over-represented in EASE analysis comparing *Il10* ^−/−^ and C57 mice at 7 or 12 weeks of age. Heat map represents the expression levels of all differentially expressed genes in the significant GO categories (EASE score <0.05 and *q* < 0.05). The groups are clearly defined within their clusters and differences in gene expression are seen for 12-week-old *Il10* ^−/−^ mice relative to 7-week-old *Il10* ^−/−^ mice and 12-week-old C57 mice.

Seven and 12 weeks of age were chosen to assess changes in colon gene and protein expression because these two time points represented no inflammation (7 weeks of age) in most of the mice (two mice with signs of inflammation were excluded) and moderate inflammation (12 weeks of age) in *Il10* ^−/−^ mice. Pathway and network analysis using IPA was conducted on the transcriptome and proteome data. The transcriptome data were also subjected to GO analysis using EASE to confirm and further support the IPA analysis. As expected, at the gene level, more changes were observed at 12 weeks than at 7 weeks of age in *Il10* ^−/−^ mice compared to C57 mice of the same age, with genes mostly being up-regulated at each time point in the colon of *Il10* ^−/−^ mice. Those gene changes are illustrated in Figure 3 which shows the genes over-represented in the EASE analysis when comparing *Il10* ^−/−^ and C57 mice at 7 and 12 weeks of age. The mean expression of selected genes obtained by qRT-PCR mostly confirmed the changes in expression levels from the microarray analysis. The changes in expression levels of *Ces2*, *Il1B*, *Igfbp5,* and *Srebf1* genes became significant in the colon of 7-week-old and 12-week-old *Il10* ^−/−^ mice, respectively by using the more sensitive qRT-PCR analysis (Table 3). 

**Figure 4:**
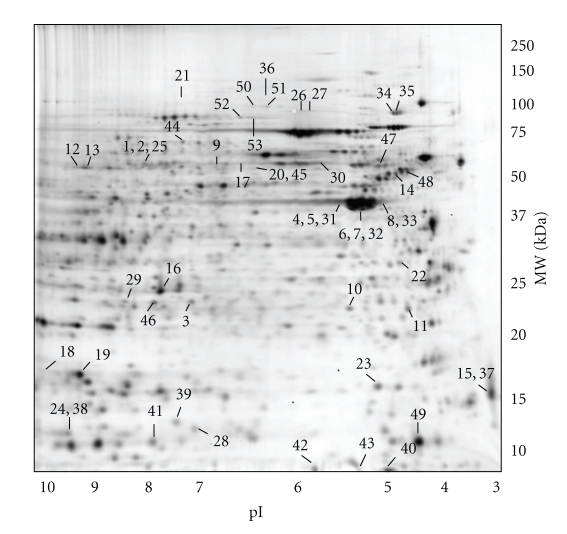
2D-DIGE gel representing differentially expressed proteins identified in the colon tissue of *Il10* ^−/−^ mice compared to C57 mice at 7 and 12 weeks of age, respectively. Protein annotations are shown in Table 4. The approximate isoelectric point (pI) and molecular weight (MW) in kDa are given on the *x*- and *y*-axes, respectively.

Only four consistent protein expression changes were identified at 7 weeks of age in a direct comparison of *Il10* ^−/−^ versus C57 mice across the pooled biological replicates (pool 1 and pool 2), with two decreased and two increased in expression levels. Of these, only two met the threshold criteria and were visible after staining for spot picking and subsequent identification. At 12 weeks of age, 44 consistent protein expression changes were observed in *Il10* ^−/−^ versus C57 mice across the pooled biological replicates (pool 1 and pool 2) with 22 decreased and 22 increased in expression levels. Here, 42 protein expression changes met the threshold criteria and were selected for subsequent identification. The combined 44 spot-features identified over the two time points represented 40 unique proteins (excluding multiple isoforms due to post-translational modifications) and are shown in the gel image depicted in Figure 4, and listed in Table 4. In seven cases, two or more proteins or protein isoforms were identified in the same 2D gel spot-feature. 

Several of these biological functions and metabolic and signaling pathways have previously been shown to be governed by PPAR*α* after ligand-induced activation, including fatty acid-, lipid and amino acid metabolism, cell cycle, immune response, and cell death both in the small intestine [27] and colon [17].

### 3.4. Gene Ontology, Network/Function, and Pathway Analysis of Colonic Genes and Proteins of *Il10* ^−/−^ and C57 Mice at 7 Weeks of Age

Genes differentially expressed at 7 weeks in the colon of *Il10* ^−/−^ compared to C57 mice were classified into 1493 GO categories. Only 21 of these categories were over-represented based on an EASE score <0.05 and *q* < 0.05; these are listed in Table 5. The EASE analysis of gene expression indicated that several biological processes were over-represented such as antigen presentation, carbohydrate, and lipid metabolism for energy utilization and steroid metabolism. Over-represented functional categories included MHC class II receptor activity. Most of the colonic genes in these GO categories showed up-regulated expression in *Il10* ^−/−^ compared to C57 mice at 7 weeks of age.

In IPA, 50 networks were generated from the genes differentially expressed in the colon of *Il10* ^−/−^ mice relative to C57 mice at 7 weeks of age. The themes of the five highest scoring networks (*P* < 0.05 using Fisher's exact test) which encompassed the highest number of differentially expressed genes in the transcriptome dataset were cancer, cell cycle, growth, proliferation, and death and cell-mediated immune response (Table 6). As there were only two proteins (adenylate cyclase-associated protein 1 and glutamate dehydrogenase 1) that had lower abundance, and one protein (peroxiredoxin) with higher abundance between *Il10* ^−/−^ and C57 mice (both at 7 weeks of age), no network analysis was carried out for protein in IPA. 

The most significantly regulated pathways in IPA containing most of the differentially expressed genes (20%) included antigen presentation (MHC class II genes such as *Hla*-*Dm*, *Hla*-*Dq*, and *Hla*-*Dr* members) and interferon signaling pathway for which the expression levels of genes were mostly increased (Table 7).

### 3.5. Gene Ontology, Network/Function, and Pathway Analysis of Colonic Genes and Proteins of *Il10* ^−/−^ and C57 Mice at 12 Weeks of Age

At 12 weeks, differentially expressed genes were classified into 1570 GO categories and 32 of these categories were significantly over-represented with an EASE score <0.05 and *q* < 0.05 and are listed in Table 8. Genes in biological process categories associated with defense response to biotic (e.g., pathogen) stimulus or stress were the most over-represented among the up-regulated genes, followed by humoral and innate immune response and antigen presentation. Genes assigned to protein activation, such as post-translational changes targeting membrane proteins, were also over-represented, and were mostly up-regulated in *Il10* ^−/−^ compared to C57 mice.

In IPA, 48 networks were generated from the colonic genes differentially expressed in *Il10* ^−/−^compared to C57 mice at 12 weeks of age. In Table 6, the top 3 biological functions for the most significant transcriptomic and proteomic networks are shown. These transcriptomic and proteomic networks share the following biological functions: cell-mediated immune response is represented in transcriptome 4 and proteome 1, cancer is represented in transcriptome 2 and proteome 3, and cell-to-cell signaling and interaction is represented in transcriptome 1 and 4. Thus, the theme linking transcriptomic and proteomic networks is cell migration and changes in tissue structure (implications for actin cytoskeleton dynamics) as well as cell death with associated inter- and intracellular signaling and initiation of the immune response.

The most significantly regulated pathways in IPA featuring most of the differentially expressed genes (20–40%) included signaling processes (Table 7). The signaling pathways included antigen presentation pathway (MHC class I, e.g., *Hla* members and MHC class II, e.g., *Hla-Dm*, *Hla-Dq*, *Hla-Dr* members, genes); graft-versus-host disease signaling and allograft rejection signaling (e.g., *Il1B*, *Tnfa*), interferon signaling (e.g., *Ifng*), role of RNA-dependent protein kinase (PKR) in interferon induction and antiviral response (e.g., *Bcl*), dendritic cell maturation and complement system, and the genes in those pathways were mostly up-regulated.

## 4. Discussion

This study characterizes colitis onset and progression at the histopathological, transcriptome, and proteome level in bacterially inoculated *Il10* ^−/−^ mice (C57BL/6J background). Here, we show that most *Il10* ^−/−^ mice developed moderate colitis already by 8.5 weeks of age and severe colitis peaking between 10 and 12 weeks of age when compared to 7-week-old *Il10* ^−/−^ mice. The nature of colon inflammation was similar to that displayed by conventionally housed *Il10* ^−/−^ mice on the same genetic background not subjected to bacterial inoculation and represented features characteristic of human CD [16]. Several aspects of innate and adaptive immune responses were affected at the gene expression level before (7 weeks of age) and after (12 weeks of age) colitis onset in *Il10* ^−/−^ mice. These findings agree with the inflammatory and immune responses observed in CD patients who have a defective interaction between their innate mucosal immune system and luminal bacteria [28]. This study focuses on those pathways linking the gene and protein expression changes such as cytoskeletal rearrangement, cell migration, and innate immunity that underlie colitis development in this *Il10* ^−/−^ mouse model. Since PPAR*α* has previously been identified as a potential key mediator in inflammation [17], the findings of this study showed that *Ppara* is involved in signaling processes before and after colitis onset likely to control colitis. Further, a potential link between PPAR*α* and IL10 is suggested.

### 4.1. Change in Transcriptomic Profile with Colitis

Increased expression levels of membrane-bound *Tlr2* and *Tlr9* genes in the colon of 12-week-old *Il10* ^−/−^compared to C57 mice were consistent with their reported function. Peptidoglycan and lipoproteins from the cell wall of commensal bacteria are recognized by TLR2 [28]. The underlying abnormal response between the innate immune system to bacterial structures is mediated via TLR and other pattern-recognition receptors which then regulate antigen-specific adaptive immune responses [29]. Dendritic cells, an important cellular component of the mucosal innate immune system, sample intestinal bacteria through pattern recognition with TLR [30]. The recognition of bacterial structures by TLR2 can lead to activation of NF*κ*B-MAPK pathways and interferon regulatory factor family members [31]. This activation in turn induces differentiation of cells producing pro-inflammatory cytokines, such as IFN*γ* and IL17, responsible for CD-like inflammation [28]. Increased mRNA abundance of the *Tlr9* gene in 7-week and 12-week-old *Il10* ^−/−^ mice compared to C57 mice may be associated with signaling events in order to maintain or reestablish colonic homeostasis, respectively. Lee et al. [31] reported distinct transcriptional responses of TLR9 activation through apical and basolateral surface domains to maintain colonic homeostasis and regulate tolerance and inflammation. Basolateral TLR9 activation of intestinal epithelial cells leads to NF*κ*B signaling, whereas apical TLR9 stimulation prevented NF*κ*B activation. These TLR-dependent innate immune responses seemed to be important in the *Il10* ^−/−^ mouse colitis model in order to adapt to luminal bacteria and their antigens. 

The gene network analysis in our previous study linked genes involved in inflammatory and immune response, tryptophan and xenobiotic metabolism, and antigen presentation [17]. Several genes of these pathways were also differentially expressed mostly in the 12-week-old *Il10* ^−/−^ mice. The higher expression levels of MHC class I and class II genes in the colon of *Il10* ^−/−^ compared to C57 mice at 12 weeks were likely to be associated with mounting antigen-specific adaptive immune responses to bacterial invasion. The expression level of *Ido1*, a gene involved in tryptophan catabolism, was increased at both 7 and 12 weeks of age in colitic *Il10* ^−/−^ mice. High expression level of the anti-inflammatory enzyme IDO was observed in intestinal biopsies from CD patients [32]. The pro-inflammatory cytokine genes *Ifng* and *Tnfa*, known as potent inducers of INDO protein activity [33], had higher mRNA abundance only in the inflamed colon of 12-week-old *Il10* ^−/−^ mice. Elevated levels of IL10 and TGFB have also been found in CD patients, and it was suggested that this modulated the immune response and caused activation of B lymphocytes [32]. The increased expression levels of tryptophan (e.g., *Ido1*) and antigen presentation (e.g., *Hla* class II members) genes in the 12-week-old *Il10* ^−/−^ mice might be linked to the restoration of tolerance towards bacterial antigens. In 7-week-old non-inflamed *Il10* ^−/−^ mice, increased *Ido1* gene expression might also be important in the early response to commensal bacteria preceding colitis due to the deficiency of the *Il10* gene. Increased mRNA abundance of genes involved in the complement activation, antigen presentation, and B-cell receptor signaling in 12-week-old *Il10* ^−/−^ mice is in agreement with functional implications of PPAR*α* activation. PPAR*α* has been associated with the innate immune response of the small intestine using Ppara^−/−^and wildtype mice, and its pharmacological activation inhibited complement activation, antigen presentation, and B-cell receptor signaling [27].

### 4.2. Change in Proteomic Profile with Colitis

Proteins differentially expressed in inflamed *Il10* ^−/−^ mice were involved in cytoskeletal rearrangement, cell-mediated immune response, and pathogen-influenced signaling. The cytoplasmic actin gamma 1 and smooth muscle actin gamma 2 proteins (ACTG1 and ACTG2) were less abundant in the colon of *Il10* ^−/−^compared to C57 mice at 12 weeks of age. Pro-inflammatory cytokines and bacteria can modify tight junctions interconnecting intestinal epithelial cells via the actin cytoskeleton so disruption of actin leads to disruption of tight junctions and loss of barrier function with increased paracellular permeability [34]. This is further supported by a study reporting decreased actin-binding gene expression levels in peripheral blood mononuclear cells of IBD patients suggesting that disruption of the actin cytoskeleton might contribute to CD pathogenesis in humans [35]. The ACTG2 protein was also decreased in expression at the gene level in the 12-week-old *Il10* ^−/−^ mice compared with C57 mice, but the *Actg2* gene remained unchanged in *Il10* ^−/−^ mice at 7 weeks of age when the intestinal barrier was presumably still intact.

Actin-binding proteins including gelsolin (GSN) involved in the regulation of actin-based motility by calcium-dependent Rho-family GTPases (e.g., RhoA, Rac1 and Cdc42) were differentially expressed in the colon of *Il10* ^−/−^ mice at 12 weeks of age. Rho-family proteins share growth-promoting and anti-apoptotic functions, regulation of gene expression through activation of signaling molecules, for example, NF*κ*B [36]. They can promote actin cytoskeleton reorganization, but Rho-family proteins differ in their effect on cell movement; for example, RhoA induces the formation of stress fibers and focal adhesions [36]. Rho GDP dissociation inhibitor (GDI) beta (Rho GDI*β*) protein, which is encoded by the *Arhgdib* gene (mRNA abundance was increased in 12-week-old *Il10* ^−/−^ mice), regulates RhoGTPase activity by inhibiting GDP dissociation to leave RhoGTPases inactive [37]. This suggests that in the inflamed colon of those *Il10* ^−/−^ mice, Rho protein activity is being inhibited by Rho GDI*β* and thus inhibits actin cytoskeleton reorganization. Enhanced T-cell migration as a consequence of the inflammatory state of the intestine is maintained by pro-inflammatory cytokines and has been associated with increased expression levels of GSN protein in smooth muscle cells in the small intestine of CD patients [38]. However, the expression level of GSN protein and *Gsn* gene was reduced in the colon of 12-week-old *Il10* ^−/−^ mice which might reflect a declining inflammatory cell movement. This is supported by the decreased inflammatory lesions in the colon of *Il10* ^−/−^ mice from 12 weeks to 14 weeks of age and might be associated with reduced inflammatory cell migration. Gelsolin-null mice have shown reduced neutrophil and fibroblast movement [39]. 

The actin cytoskeleton is a flexible system that is built up or broken down depending on antigen recognition, cell polarization, and cell adhesion or cell migration. Actin-binding proteins are involved in surface receptor clustering during T-cell activation and migration to dynamic cytoskeletal rearrangements at the interface between T-cells and antigen presenting cells [40]. A study using an *in vitro* model of simulated ischemia-reperfusion showed an involvement of Rho-kinase-dependent cytoskeletal rearrangement in apoptosis initiation [41]. In the colon of the 12-week-old *Il10* ^−/−^ mice, Rho GDI*β* may have inhibited or delayed actin rearrangement, and in part attenuated apoptosis, anti-apoptosis genes such as *Bcl2A1* gene was increased in expression. BCL3, a component of the cellular cytokine-induced inflammatory signaling cascade, has been shown to interact cooperatively with PPARGC1*α* to activate estrogen-related receptors and PPAR*α* resulting in increased expression of target genes involved in cellular energy metabolism [42]. Although *Bcl3* mRNA abundance was increased, *Ppara* and *Ppargc1a* gene expression levels decreased in 7-week, and 12-week-old *Il10* ^−/−^ mice which imply an impaired energetic adaptation of the colonocytes to bacterial- or cytokine-induced cellular stress at an early and late stage, respectively. This is supported by findings from others that the failure of regulatory mechanism in *Il10* ^−/−^ mice under developing colitis primes the epithelium towards energy deficiency and uncontroled cellular stress leading to tissue damage [15]. IL10 and also PPAR*α* seem to be important in this regulatory mechanism.

The thioredoxin (TXN) protein, known to function as an antioxidant in the maintenance of cellular redox homeostasis, was more highly expressed in 12-week-old *Il10* ^−/−^ mice compared with C57 mice. Thioredoxin has previously been identified as a PPAR*α* target gene and a negative autoregulation of PPAR*α* activity by thioredoxin was suggested as a novel mechanism for controling PPAR*α* activities and PPAR*α*-related physiological or pathological processes [43]. The finding of the present study may also be explained by a functional down-regulation of normal PPAR*α* activities such as lipid and inflammatory regulation through the thioredoxin-mediated negative autoregulation of PPAR*α* transcriptional activity.

### 4.3. *Il10* ^−/−^ Colitis Model and PPAR*α* Signaling

Several of the gene expression changes in colitic *Il10* ^−/−^ mice at 12 weeks of age were related to PPAR*α* signaling. A regulatory role for PPAR*α* in inflammation was first shown by the prolonged duration of inflammation in Ppara^−/−^ mice [18], and network analysis identified *Ppara* as a key mediator gene decreased in expression during colitis in *Il10* ^−/−^ compared to C57 mice [17]. Furthermore, PPAR*α* and also PPAR*γ* ligands exerted anti-inflammatory effects in inflammatory disease models. For example, administration of PPAR*α* (bezafibrate) and PPAR*γ* (troglitazone) ligands reduced dextran sulphate sodium-induced colitis and cell proliferation in colonic mucosa [19], PPAR*α* ligand fenofibrate decreased expression levels of pro-inflammatory *Ifng* and *Il17* genes in *Il10* ^−/−^ mice [20], PPAR*γ* ligand rosiglitazone delayed colitis onset in *Il10* ^−/−^ mice [7], and fenofibrate prevented the progression of autoimmune myocarditis in rats through increased cardiac *Il10* mRNA levels [44]. 

PPAR*α* is expressed in colonic immune and epithelial cells where it acts in an anti-inflammatory manner upon activation by a PPAR*α* ligand [20]. In the present study, the expression level of the *Ppara* gene was decreased in inflamed and non-inflamed colons of *Il10* ^−/−^ mice when compared to C57 mice without exogenous ligand-mediated PPAR*α* activation. It is known that PPAR*α* governs inflammation mainly by down-regulating gene expression such as acute phase response genes [45]. PPAR*α* (mRNA and protein) expression levels were decreased in colorectal cancer in the *A* *P* *C* ^Min /+^ mouse model of familial adenomatous polyposis compared with matched non-malignant tissue [46]. Ppara^−/−^ mice show enhanced susceptibility dinitrobenzene sulfonate (DNBS)-induced colitis [47]. PPAR*α* ligands reduce colitis in chemically induced and genetic (including *Il10* ^−/−^ mice) models of colitis [19, 20], whereas the absence of PPAR*α* abolishes the protective effect of the PPAR*α* ligand WY14643 in DNBS-induced colitis [47]. Thus, activation of PPAR*α* appears to be a target for controling colitis including in this inoculated *Il10* ^−/−^ mouse model. The decreased *Ppara* gene expression level in 7-week-old *Il10* ^−/−^ mice suggests that immune cells might have been activated due to increased bacterial invasion and a lack of regulatory mechanisms through *Il10* gene deficiency, even though histopathological signs of colitis were not clearly evident yet. It has been reported that PPAR*α* expression in murine lymphocytes is rapidly decreased following T-cell activation suggesting a role for PPAR*α* in immune cells [48].

The anti-inflammatory effect of fenofibrate by delaying colitis onset and progression in *Il10* ^−/−^ mice indicates that IL10 is not required in PPAR*α* signaling [20]. In contrast, another study emphasized the stimulation of the IL10 pathway in rats treated with fenofibrate to suppress myocarditis [44]. This implicates a potential PPAR*α*-dependent regulation of IL10. So far, it has been shown that rosiglitazone induced IL10 production from human mature dendritic cells and CD4+ T-cells [49]. This effect was PPAR*γ*-dependent due to a functional PPAR response element for PPAR*γ* in the human IL10 promoter region.

In conclusion, the findings from this study identified distinct colonic gene and protein expression profiles for 7- and 12-week-old *Il10* ^−/−^ mice. Gene and protein expression data linked actin cytoskeleton dynamics, innate immunity, and apoptosis and suggested a delayed remodeling process in the colitic *Il10* ^−/−^ mice. PPAR*α* might be one of the key mediators in these signaling processes before and after colitis onset in *Il10* ^−/−^ mice. Ligand-mediated PPAR*α* activation and IL10 production may be important for the anti-inflammatory effects of PPAR*α* observed in inflammatory disease models and possibly in the resolution of inflammatory disease in humans. Confirmation of a role for PPAR*α* activation in intestinal IL10 production, and clarification of the mechanism by which this occurs, warrants further investigation. 

## Figures and Tables

**Table 1:** Body weight and dietary intake of *Il10* ^−/−^ and C57 mice at 7, 8.5, 10, 12, and 14 weeks of age.

Weeks of age	Body weight (g)		Dietary intake (g)	
	C57 mice	*Il10* ^−/−^ mice	C57 mice	*Il10* ^−/−^ mice
7	18.8 ± 0.4	18.2 ± 0.8	3.4 ± 0.1	3.3 ± 0.2
8.5	20.3 ± 0.9	19.0 ± 0.3	3.6 ± 0.1	3.9 ± 0.3
10	22.5 ± 0.6	19.9 ± 0.4*	3.7 ± 0.0	3.9 ± 0.1
12	24.8 ± 0.7	21.0 ± 0.8*	3.6 ± 0.1	3.5 ± 0.2
14	25.3 ± 0.4	19.8 ± 0.7*	3.6 ± 0.1	3.5 ± 0.3

Data shown as mean ± standard error of mean (SEM) per group of mice sacrificed. Body weight was measured thrice weekly and dietary intake was estimated and adjusted daily to equal the mean amount of food consumed by *Il10* ^−/−^ mice on the previous day to ensure similar intakes between C57 and *Il10* ^−/−^ mice. *P < 0.05 comparing *Il10* ^−/−^ versus C57 mice of the same age.

**Table 2:** Time of onset and incidence of colon inflammation in *Il10* ^−/−^ mice.

Weeks of age	7	8.5	10	12	14
Number of mice sampled					
*Il10* ^−/−^ mice (total 28^1^)	5	6	6	6	5
C57 mice (total 30)	6	6	6	6	6
Number of mice with inflamed colon^2^					
*Il10* ^−/−^ mice	2	4	6	6	4
C57 mice	0	0	0	0	0
Incidence of colon inflammation (%)					
*Il10* ^−/−^ mice	40	67	100	100	80
C57 mice	0	0	0	0	0

^1^Two *Il10* ^−/−^ mice died during the study due to unknown causes. ^ 2^Mice were regarded as inflamed when their total colon histological injury score (HIS) was >3 (4–6 moderate inflammation, ≥7 severe inflammation). All C57 mice had a total colon HIS ≤ 3.

**Table 3:** Validation of gene expression results from microarray analysis using qRT-PCR.

Gene symbol	Fold change microarray		Fold change qRT-PCR *Canx *	
	*Il10* ^−/−^ versus C57 7 weeks	*Il10* ^−/−^ versus C57 12 weeks	*Il10* ^−/−^ versus C57 7 weeks	*Il10* ^−/−^ versus C57 12 weeks
*Abcb1A*	−3.4	−2.8	−5.2	−4.9
*Aldh1A1*	−1.5*	−3.0	−1.5^#^	−3.4
*Ces2*	−1.8*	−3.1	−2.9	−5.6
*Fabp2*	−1.9	−5.6	−1.5^#^	−9.8
*Igfbp5*	−1.5*	1.6*	−1.9^#^	2.8
*Il1B*	1.8*	6.4	3.4	8.5
*Mmp13*	1.0*	1.8	2.7^#^	9.9
*Ppara*	−2.0	−1.7	−5.6	−3.8
*Srebf1*	1.0*	1.0*	1.2^#^	1.8
*Sult1A1*	−3.2	−3.1	−3.6	−3.3

*Canx* (calnexin) reference gene used to to normalize the data; *microarray result was not significantly different using moderated *t*-statistics and false discovery rate (FDR) control in limma; genes that satisfied the criterion of FC ≥ 1.5 and *q* < 0.05 were considered to be significantly different; ^#^qRT-PCR result was not significantly different using ANOVA.

**Table 4:** Proteins differentially expressed in the colon of *Il10* ^−/−^ mice compared to C57 mice at 7 and 12 weeks of age.

Spot	Accession number	Protein description	Gene (MGI)	Theoretical M_r_ (kDa)	Theoretical pI	No. peptides matched	Sequence coverage %	SEQUEST P (pro)	Fold change
7 weeks									
1	gi*∣*157951604	CAP, adenylate cyclase-associated protein 1	*Cap1*	51.5	7.29	2	4.2	9.50E-05	−2.9
2	gi*∣*6680027	glutamate dehydrogenase 1	*Glud1*	61.3	7.96	3	6.1	2.85E-10	−2.9
3	gi*∣*6671549	peroxiredoxin 6	*Prdx6*	24.8	5.96	5	27.2	3.99E-08	3.4

12 weeks									
4	gi*∣*6752954	actin, gamma, cytoplasmic 1	*Actg1*	41.8	5.20	10	38.9	7.54E-12	−3.2
5	gi*∣*6671507	actin, alpha 2, smooth muscle, aorta	*Acta2*	42.0	5.12	3	13.3	2.13E-06	−3.2
6	gi*∣*6752954	actin, gamma, cytoplasmic 1	*Actg1*	41.8	5.20	9	30.7	2.76E-10	−7.4
7	gi*∣*148666671	actin, gamma 2, smooth muscle, enteric	*Actg2*	41.9	5.20	4	20.7	1.50E-06	−7.4
8	gi*∣*6752954	actin, gamma, cytoplasmic 1	*Actg1*	41.8	5.20	6	15.7	7.93E-07	−2.9
9	gi*∣*21312260	aldehyde dehydrogenase 1 family, member B1	*Aldh1b1*	57.5	6.61	8	16.6	2.88E-09	−3.3
10	gi*∣*160333304	apolipoprotein A-I	*Apoa1*	30.6	5.57	4	16.7	5.56E-12	2.4
11	gi*∣*33563236	Rho, GDP dissociation inhibitor (GDI) beta	*Arhgdib*	22.8	4.80	4	22.5	4.11E-13	2.9
12	gi*∣*6680748	ATP synthase, H+ transporting, mitochondrial F1 complex, alpha subunit, isoform 1	*Atp5a1*	59.7	9.53	15	35.1	3.40E-12	−2.0
13	gi*∣*6680748	ATP synthase, H+ transporting, mitochondrial F1 complex, alpha subunit, isoform 1	*Atp5a1*	59.7	9.53	12	27.8	1.72E-12	−2.1
14	gi*∣*31980648	ATP synthase, H+ transporting mitochondrial F1 complex, beta subunit	*Atp5b*	56.3	5.07	16	41.8	5.47E-12	−2.1
15	gi*∣*6753244	calmodulin 1	*Calm1*	16.8	3.93	3	32.9	6.21E-11	5.0
16	gi*∣*145301561	carbonic anhydrase 1	*Car1*	28.3	6.49	7	36.4	4.32E-10	2.3
17	gi*∣*126521835	chaperonin subunit 2 (beta)	*Cct2*	57.4	5.96	11	26.7	2.76E-09	−2.2
18	gi*∣*6680924	cofilin 1, non-muscle	*Cfl1*	18.5	8.20	4	18.8	8.89E-09	2.9
19	gi*∣*6680924	cofilin 1, non-muscle	*Cfl1*	18.5	8.20	4	18.8	1.28E-09	2.5
20	gi*∣*6755522	evolutionarily conserved signalling intermediate in Toll pathway	*Ecsit*	49.7	6.14	2	4.1	1.54E-05	−2.3
21	gi*∣*33859482	eukaryotic translation elongation factor 2	*Eef2*	95.3	6.30	10	13.4	8.98E-12	−5.8
22	gi*∣*123261836	EF hand domain containing 2	*Efhd2*	26.8	4.92	5	24.2	3.42E-08	2.0
23	gi*∣*31712036	eukaryotic translation initiation factor 5A	*Eif5a*	16.8	4.94	3	20.8	9.81E-12	2.2
24	gi*∣*14149635	fatty acid binding protein 4, adipocyte	*Fabp4*	14.6	8.74	5	48.5	1.28E-08	2.9
25	gi*∣*6680027	glutamate dehydrogenase 1	*Glud1*	61.3	7.96	10	20.1	7.64E-11	−2.9
26	gi*∣*123123547	gelsolin	*Gsn*	85.9	5.78	5	10.4	5.61E-07	−2.3
27	gi*∣*123123547	gelsolin	*Gsn*	85.9	5.78	3	4.4	2.19E-07	−2.5
28	gi*∣*56238582	histidine triad nucleotide binding protein	*Hint1*	13.8	6.41	2	16.7	1.39E-10	2.1
29	gi*∣*62024583	Cr1 protein	*Igkv1-117*	26.3	7.97	2	11.3	2.05E-09	2.2
30	gi*∣*114145561	keratin complex 2, basic, gene 8	*Krt8*	54.5	5.59	16	39.6	1.20E-11	−2.0
31	gi*∣*123236748	keratin complex 1, acidic, gene 19	*Krt19*	44.5	5.15	6	12.9	6.39E-10	−3.2
32	gi*∣*123236748	keratin complex 1, acidic, gene 19	*Krt19*	44.5	5.15	13	45.4	1.82E-08	−7.4
33	gi*∣*123236748	keratin complex 1, acidic, gene 19	*Krt19*	44.5	5.15	9	25.3	8.96E-10	−2.9
34	gi*∣*40556608	heat shock protein 1, beta	*Hsp90ab1*	83.2	4.82	15	22.1	2.47E-10	−2.8
35	gi*∣*40556608	heat shock protein 1, beta	*Hsp90ab1*	83.2	4.82	17	25.3	1.01E-11	−2.1
36	gi*∣*26984237	mitochondrial ATP-dependent protease Lon	*Lonp1*	10.6	6.20	4	4.0	5.94E-05	−3.0
37	gi*∣*149266035	PREDICTED: similar to non-muscle myosin alkali light chain	*Myl6*	13.0	4.42	3	34.5	7.28E-06	5.1
38	gi*∣*6755040	profilin 1	*Pfn1*	15.0	8.43	6	55.7	1.42E-12	2.9
39	gi*∣*51772387	PREDICTED: similar to 40S ribosomal protein S12	*Rsp12*	14.5	6.94	2	12.1	4.18E-04	2.1
40	gi*∣*33859624	S100 calcium binding protein A4	*S100a4*	11.7	5.07	2	17.8	1.43E-04	2.4
41	gi*∣*6677837	S100 calcium binding protein A9 (calgranulin B)	*S100a9*	13.0	6.76	4	36.3	7.54E-06	4.0
42	gi*∣*18314646	S100 calcium binding protein A11 (calgizzarin)	*S100a11*	11.1	5.15	1	16.3	1.65E-04	5.1
43	gi*∣*18314646	S100 calcium binding protein A11 (calgizzarin)	*S100a11*	11.1	5.15	1	16.3	3.53E-05	3.2
44	gi*∣*14389431	stress-induced phosphoprotein 1	*Stip1*	62.5	6.39	7	11.6	1.31E-05	−3.9
45	gi*∣*27754031	sorting nexin 6	*Sx6*	45.0	5.47	3	7.14	2.62E-05	−2.3
46	gi*∣*226958349	triosephosphate isomerase 1	*Tpi1*	26.7	7.07	3	19.3	6.59E-09	2.1
47	gi*∣*6678469	tubulin, alpha 1C	*Tuba1c*	50.1	4.83	12	34.5	1.26E-11	−2.0
48	gi*∣*7106439	tubulin, beta 5	*Tubb5*	49.7	4.64	9	34.7	1.30E-11	−2.2
49	gi*∣*123210316	thioredoxin	*Txn*	11.7	4.64	4	31.4	4.27E-07	2.5
50	gi*∣*148877507	villin 1	*Vil1*	92.7	5.66	13	18.7	8.57E-09	−3.2
51	gi*∣*148877507	villin 1	*Vil1*	92.7	5.66	20	26.4	4.52E-08	−4.6
52	gi*∣*83921618	villin 2	*Vil2*	69.4	5.76	7	14.8	3.15E-09	−2.4
53	gi*∣*83921618	villin 2	*Vil2*	69.4	5.76	8	15.9	1.99E-08	−2.6

**Table 5:** Categories of genes with expression increase of 2-fold or more in *Il10* ^−/−^ mice compared to C57 mice at 7 weeks of age using EASE.

System	Gene category	EASE score	FDR
GO Cellular Component	spindle cell	3.91E-04	<0.001
GO Molecular Function	MHC class II receptor activity	1.25E-03	<0.001
GO Biological Process	antigen processing, exogenous antigen via MHC class II	1.37E-03	<0.001
GO Biological Process	antigen presentation, exogenous antigen	1.37E-03	<0.001
GO Biological Process	alcohol metabolism	2.27E-03	<0.001
GO Biological Process	glucose metabolism	4.37E-03	<0.001
GO Biological Process	main pathways of carbohydrate metabolism	6.39E-03	<0.001
GO Biological Process	protein targeting	7.76E-03	<0.001
GO Biological Process	steroid biosynthesis	1.02E-02	<0.001
GO Biological Process	steroid metabolism	1.09E-02	<0.001
GO Biological Process	sterol biosynthesis	1.29E-02	<0.001
GO Cellular Component	microtubule cytoskeleton	1.75E-02	<0.001
GO Biological Process	sterol metabolism	1.82E-02	<0.001
GO Biological Process	carbohydrate metabolism	1.89E-02	<0.001
GO Biological Process	hexose metabolism	1.92E-02	<0.001
GO Molecular Function	oxidoreductase activity, acting on CH-OH group of donors	1.96E-02	<0.001
GO Biological Process	energy derivation by oxidation of organic compounds	2.47E-02	<0.001
GO Biological Process	catabolism	2.54E-02	<0.001
GO Cellular Component	soluble fraction	2.82E-02	<0.001
GO Molecular Function	oxidoreductase activity, acting on the CH-OH group of donors, NAD or NADP as acceptor	2.92E-02	<0.001
GO Biological Process	monosaccharide metabolism	3.13E-02	<0.001

**Table 6:** Proteome and transcriptome network analysis for expression profiles from the colon of 7- and 12-week-old *Il10* ^−/−^ mice compared to C57 mice using IPA.

Network	Top functional categories
*Il10* ^−/−^ versus C57 mice at 7 weeks of age	
Transcriptome 1	Cancer, Dermatological disease and conditions, Endocrine system development and function
Transcriptome 2	Cardiovascular system development and function, Cancer, Tissue development
Transcriptome 3	Cancer, Cell death, Reproductive system disease
Transcriptome 4	Cell cycle, Cell-mediated immune response, Cancer
Transcriptome 5	Cellular growth and proliferation, Hematological system development and function, Hematopoiesis

*Il10* ^−/−^versus C57 mice at 12 weeks of age	
Proteome 1	Tissue morphology, Cellular movement, Cell-mediated immune response
Proteome 2	Cell death, Hematological disease, Immunological disease
Proteome 3	Cancer, Tumour morphology, Cellular development
Proteome 4	Cardiovascular disease, Gene expression, Molecular transport
Transcriptome 1	Antigen presentation, Cell morphology, Cell-to-cell signaling and interaction
Transcriptome 2	Cancer, Gastrointestinal disease, Dermatological diseases and conditions
Transcriptome 3	Lipid metabolism, Small molecule biochemistry, Carbohydrate metabolism
Transcriptome 4	Inflammatory disease, Cell-to-cell signaling and interaction, Cell-mediated immune response

The top 3 biological functions for the most significant transcriptomic and proteomic networks (representing subsets of focus genes and proteins highly associated with those functions) are shown.

**Table 7:** Differentially expressed genes in the colon of *Il10* ^−/−^ mice compared to C57 mice at 7 and 12 weeks of age.

Gene symbol	Gene name	GenBank accession	*Il10* ^−/−^ versus *C57 *		*Il10* ^−/−^ versus *C57 *	
			7 weeks of age		12 weeks of age	
			FC	FDR	FC	FDR
*Actg2*	actin, gamma 2, smooth muscle, enteric	NM_007392	−1.3	0.315	−1.6	0.02
*Arhgap12*	Rho GTPase activating protein 12	NM_029277	−1.1	0.549	−1.5	<0.001
*Arhgdib*	Rho GDP dissociation inhibitor (GDI) beta	NM_007486	1.1	0.562	2.0	<0.001
*Arhgef10L*	Rho guanine nucleotide exchange factor (GEF) 10-like	AK028648	2.4	<0.001	2.3	<0.001
*Bcl2A1*	BCL2-related protein A1	NM_007534	1.6	0.001	2.7	<0.001
*Bcl3*	B-cell CLL/lymphoma 3	NM_033601	1.6	0.044	1.7	0.015
*C1Qa*	complement component 1, q subcomponent, A chain	NM_007572	1.1	0.67	1.6	0.003
*C1Qc*	complement component 1, q subcomponent, C chain	NM_007574	1.3	0.29	1.8	0.005
*C1R*	complement component 1, r subcomponent	NM_023143	1.5	0.018	2.0	<0.001
*C1S*	complement component 1, s subcomponent	NM_144938	1.3	0.059	1.9	<0.001
*C2*	complement component 2	NM_013484	1.2	0.337	2.6	<0.001
*C3*	complement component 3	NM_009778	1.8	0.015	4.5	<0.001
*C4B*	complement component 4B (Chido blood group)	NM_009780	−1.0	0.976	1.6	0.005
*Ccl5*	chemokine (C-C-motif) ligand 5 (Rantes)	NM_013653	1.1	0.7	2.3	<0.001
*Cd74*	CD74 molecule, major histocompatibility complex, class II invariant chain	NM_010545	4.0	<0.001	5.5	<0.001
*Fas*	Fas (TNF receptor superfamily, member 6)	NM_007987	1.9	<0.001	1.5	<0.001
*Gsn*	gelsolin	NM_146120	−1.8	0.006	−2.0	<0.001
*Hla-A*	major histocompatibility complex, class I, A	NM_010391	1.4	0.201	2.3	<0.001
*Hla-B*	major histocompatibility complex, class I, B	NM_008199	−1.4	0.104	1.9	<0.001
*Hla-C*	major histocompatibility complex, class I, C	NM_010380	1.4	0.005	2.3	<0.001
*Hla-Dma*	major histocompatibility complex, class II, DM alpha	NM_010386	1.5	<0.001	2.1	<0.001
*Hla-Dmb*	major histocompatibility complex, class II, DM beta	NM_010387	4.9	<0.001	6.6	<0.001
*Hla-Dqa1*	major histocompatibility complex, class II, DQ alpha 1	NM_010378	1.8	<0.001	2.9	<0.001
*Hla-Dqb2*	major histocompatibility complex, class II, DQ beta 2	NM_010379	3.1	<0.001	5.3	<0.001
*Hla-Drb1*	major histocompatibility complex, class II, DR beta 1	NM_010382	2.5	<0.001	4.5	<0.001
*Hla-G*	major histocompatibility complex, class I, G	NM_013819	1.4	0.013	2.0	<0.001
*Hmgcr*	3-hydroxy-3-methylglutaryl-Coenzyme A reductase	NM_008255	1.7	0.005	1.5	0.005
*Ido1* *(Indo)*	indoleamine 2,3-dioxygenase 1	NM_008324	6.6	<0.001	8.1	<0.001
*Ifng*	interferon, gamma	NM_008337	1.4	0.071	1.7	0.003
*Il1B*	interleukin 1, beta	NM_008361	1.8	0.211	6.4	<0.001
*Il1Rn*	interleukin 1 receptor antagonist	NM_031167	1.3	0.442	1.8	0.024
*Il10*	interleukin 10	NM_010548	1.1	0.461	1.1	0.421
*Mapk13*	mitogen-activated protein kinase 13	NM_011950	1.8	<0.001	1.7	<0.001
*Mapk9*	mitogen-activated protein kinase 9	NM_016961	1.5	0.004	1.6	<0.001
*Nfkbia*	nuclear factor of kappa light polypeptide gene enhancer in B-cells inhibitor, alpha	NM_010907	1.6	0.002	1.6	0.001
*Nfkbie*	nuclear factor of kappa light polypeptide gene enhancer in B-cells inhibitor, epsilon	NM_008690	1.5	0.001	1.7	<0.001
*Ppara*	peroxisome proliferator-activated receptor alpha	NM_011144	−2.0	<0.001	−1.7	0.001
*Ppargc1A*	peroxisome proliferator-activated receptor gamma, coactivator 1 alpha	NM_008904	−1.7	0.008	−1.4	0.052
*Tlr2*	toll-like receptor 2	NM_011905	1.1	0.857	1.9	0.001
*Tlr9*	toll-like receptor 9	NM_031178	1.9	<0.001	1.9	<0.001
*Tnf*	tumour necrosis factor (TNF superfamily, member 2)	NM_013693	1.3	0.064	1.7	<0.001
*Tnfrsf1B*	tumour necrosis factor receptor superfamily, member 1B	NM_011610	1.3	0.115	1.5	0.003
*Txn*	thioredoxin	NM_011660	−1.2	0.494	−1.0	0.932

Genes were grouped into one or more of the following IPA pathways: antigen presentation, graft versus host disease signaling, allograft rejection signaling, interferon signaling, role of PKR in interferon induction and antiviral response, dendritic cell maturation, complement system, endoplasmic reticulum stress, and steroid metabolism. Genes with fold change (FC) ≥1.5 and false discovery rate (FDR) or *q* < 0.05 were considered for pathway analysis.

**Table 8:** Categories of genes with expression increase of 2-fold or more in *Il10* ^−/−^mice compared to C57 mice at 12 weeks of age using EASE.

System	Gene category	EASE score	FDR
GO Biological Process	defence response	5.92E-14	<0.001
GO Biological Process	response to biotic stimulus	7.39E-14	<0.001
GO Biological Process	response to external stimulus	1.38E-12	<0.001
GO Biological Process	immune response	4.06E-12	<0.001
GO Biological Process	antigen presentation	4.14E-07	<0.001
GO Biological Process	antigen processing	4.52E-06	<0.001
GO Molecular Function	defence/immunity protein activity	6.14E-06	<0.001
GO Molecular Function	MHC class I receptor activity	2.67E-05	<0.001
GO Biological Process	response to pest/pathogen/parasite	1.43E-04	<0.001
GO Biological Process	physiological process	1.47E-04	<0.001
GO Molecular Function	antigen binding	2.31E-04	<0.001
GO Biological Process	response to stress	2.49E-04	<0.001
GO Biological Process	antigen presentation, endogenous antigen	1.21E-03	<0.001
GO Molecular Function	MHC class II receptor activity	1.56E-03	<0.001
GO Biological Process	antigen processing, exogenous antigen via MHC class II	1.67E-03	<0.001
GO Biological Process	antigen presentation, exogenous antigen	1.67E-03	<0.001
GO Cellular Component	extracellular space	1.75E-03	<0.001
GO Biological Process	protein targeting	2.57E-03	<0.001
GO Biological Process	humoral immune response	2.97E-03	<0.001
GO Cellular Component	plasma membrane	7.23E-03	<0.001
GO Biological Process	antigen processing, endogenous antigen via MHC class I	8.44E-03	<0.001
GO Biological Process	response to wounding	1.07E-02	<0.001
GO Molecular Function	oxidoreductase activity, acting on CH-OH group of donors	1.15E-02	<0.001
GO Molecular Function	oxidoreductase activity, acting on the CH-OH group of donors, NAD or NADP as acceptor	1.71E-02	<0.001
GO Molecular Function	hydrolase activity	1.86E-02	<0.001
GO Biological Process	collagen catabolism	2.07E-02	<0.001
GO Biological Process	posttranslational membrane targeting	2.18E-02	<0.001
GO Biological Process	innate immune response	2.22E-02	<0.001
GO Cellular Component	vesicular fraction	2.51E-02	<0.001
GO Biological Process	response to chemical substance	3.06E-02	<0.001
GO Biological Process	inflammatory response	3.06E-02	<0.001
